# Significant improvement in temporary dental rehabilitation by notable miniplate application in the hard palate for a patient with a large anterior maxillary defect: a case report/technique article

**DOI:** 10.1186/1746-160X-9-34

**Published:** 2013-11-09

**Authors:** Shigeo Ishikawa, Noriaki Kikuchi, Takehito Kobayashi, Hideyuki Yamanouchi, Hirohiko Tachibana, Mitsuyoshi Iino

**Affiliations:** 1 Department of Dentistry, Oral and Maxillofacial Surgery, Okitama Public General Hospital, 2000 Nishi-Otsuka Kawanishi town, Higashi-Okitama-gun, Yamagata 992-0601, Japan; 2 Department of Dentistry, Oral and Maxillofacial Plastic and Reconstructive Surgery, Faculty of Medicine, Yamagata University, 2-2-2 Iida-nishi, Yamagata 990-9585, Japan

**Keywords:** Dental rehabilitation, Maxillary defect, Titanium miniplate

## Abstract

The present report describes the application of a miniplate in the hard palate of a 36-year-old patient with a large anterior maxillary defect. The combination of orthodontic elastics with a titanium miniplate improved the stability of the prefabricated prosthesis. This structure retained the ointment gauze covering the wounds and maintained the facial contour. In addition, contracture deformity was prevented by insertion of the prefabricated prosthesis intraoperatively or immediately postoperatively. Furthermore, a soft diet could be ingested immediately postoperatively. The miniplate also supported the anterior part and the definitive prosthesis. This prosthesis restored adequate masticatory, deglutitive, and speech functions and maintained the facial contour with minimum overloading of the remaining teeth.

## Background

Dental rehabilitation after maxillary resection is conventionally performed with an obturator prosthesis [1-4]. The obturator should promptly restore adequate masticatory function, deglutition, speech, and acceptable facial form. However, adequate dental rehabilitation for patients with large anterior maxillary defects caused by cancer-ablative surgery is often difficult because of insufficient anterior bony support for the denture. The present report describes the application of a miniplate in the hard palate of a patient with a large anterior maxillary defect. The miniplate not only enabled immediate placement of the obturator, but also provided sufficient stability of the definitive prosthesis.

### Case presentation/technique description

**Figure 1:**
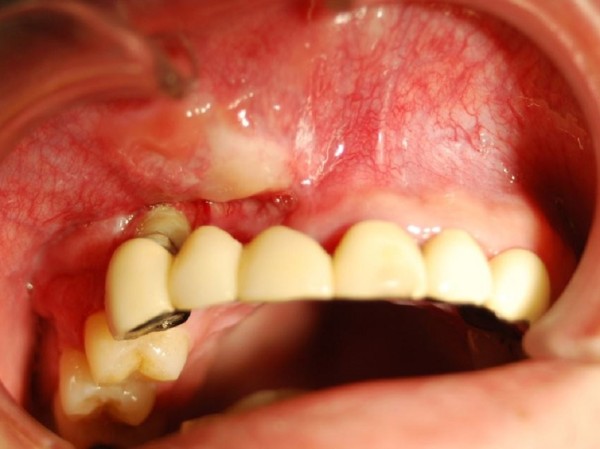
Preoperative view (oral cavity).

**Figure 2:**
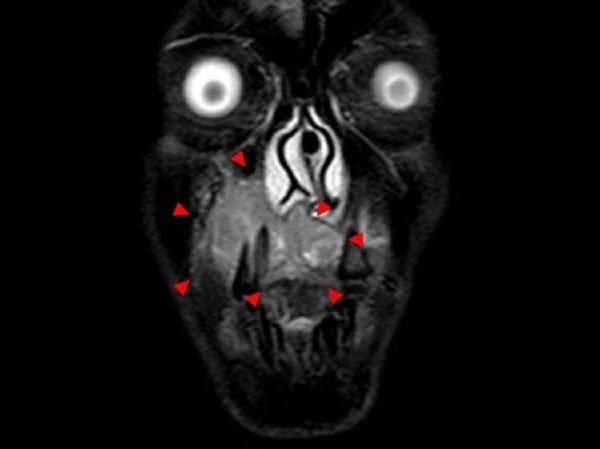
Preoperative magnetic resonance imaging shows the lesion in the right maxilla (arrows).

A 36-year-old man was referred to our clinic because of diffuse swelling of the anterior maxilla (Figure 1). The histological diagnosis was well-differentiated squamous cell carcinoma. MRI revealed a large tumor of >6 cm in diameter. Two-thirds of the hard palate and skin around the right nasal ala were involved in the tumor (Figure 2). Swelling of a right submandibular lymph node was also recognized. Preoperative intra-arterial chemotherapy and radical surgery were planned.

**Figure 3:**
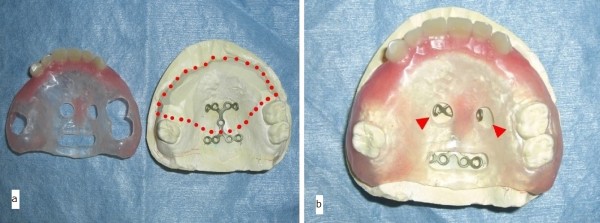
**Prefabricated prosthesis. (a)** The prefabricated prosthesis with an H-shaped titanium miniplate in the palate. The dotted line represents the planned resection boundary of the maxilla. **(b)** The tip of the miniplate extruded through the two center holes (arrows). Orthodontic elastics retained the prosthesis by securing the two free ends of the miniplate through these two holes.

The prosthesis that was to be utilized immediately postoperatively was fabricated before the surgery. This prosthesis had two holes in the central part of the palate. The two free ends of the H-shaped titanium locking plate (Compact Lock 2.0®; Depuy Synthes, Japan) were bent to protrude through the two holes. Orthodontic elastics retained the prosthesis by securing the two free ends of the H-shaped miniplate through these two holes (Figure 3a,b). The miniplate was planned to undergo fixation with 4.0-mm locking screws (Compact Lock 2.0®; Depuy Synthes, Japan).

**Figure 4:**
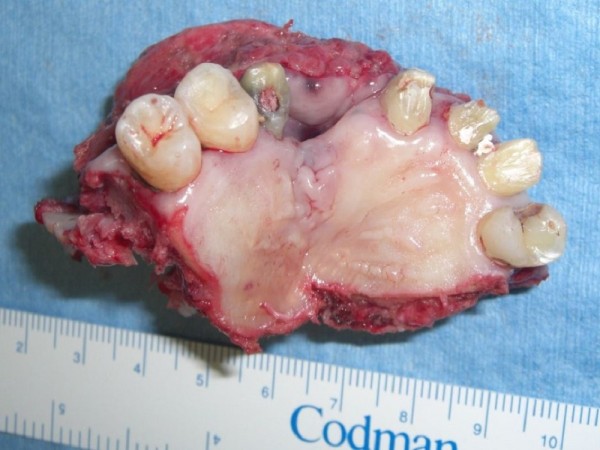
Resected specimen of the maxilla (occlusal view).

**Figure 5:**
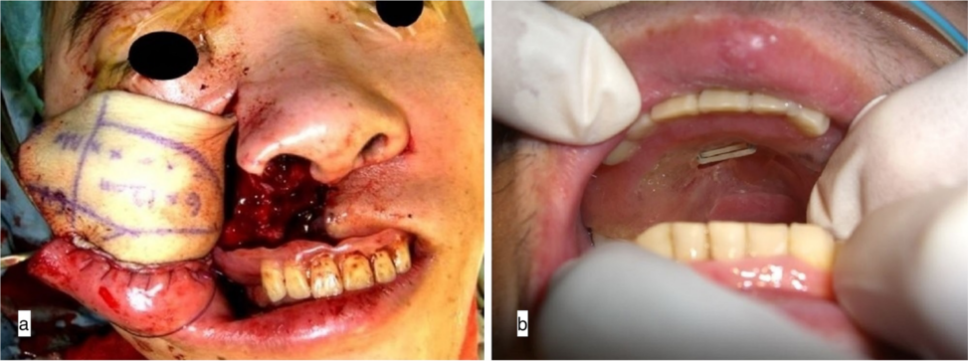
**Insertion of prefabricated prosthesis. (a)** Immediate postoperative maxillary prosthesis retained by the miniplate and orthodontic elastics (frontal view). **(b)** Prosthesis retained by the miniplate and orthodontic elastics 2 weeks after surgery (occlusal view).

Superselective intra-arterial infusion chemotherapy with cisplatin (150 mg) was performed every week for three cycles. The patient underwent tumor ablative surgery under general anesthesia. Initially, an open tracheotomy was created. After bilateral supraomohyoid neck dissection, the primary tumor was radically excised via a Diefenbach–Weber skin incision. The surgical specimen comprised the anterior two-thirds of the maxilla and nasal septum, the bilateral inferior nasal conchae, and the skin around the nasal ala and nostril of the right side (Figure 4). During the surgery, the H-shaped miniplate was fixed with three screws to the remaining hard palate. The prefabricated denture was then fitted. The two free ends of the H-shaped miniplate protruded from the two holes in the central part of the denture, and the orthodontic elastic was applied with forceps (Figure 5a,b). A prefabricated denture-based surgical obturator was fitted. The resulting surgical defect was reconstructed using a partially double-folded free radial forearm flap. This prosthesis facilitated retention of the ointment gauze covering the nasal wounds and preservation of the facial contour. With this prosthesis, the patient was able to ingest a soft diet 2 weeks after the surgery. He also learned to attach and remove the prosthesis and orthodontic elastics with forceps in the mirror by himself.

**Figure 6:**
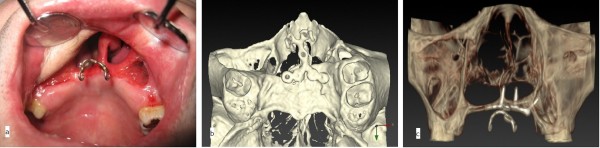
**Postoperative view of miniplate fixation. (a)** Frontal view of the titanium miniplate. **(b)** Occlusal view of a three-dimensional computed tomography (3D-CT) image with the titanium miniplate. **(c)** Frontal view of a 3D-CT image with the titanium miniplate.

**Figure 7:**
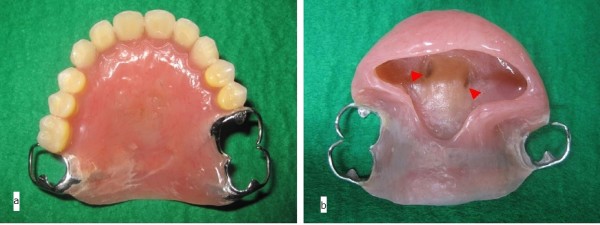
**Definitive prosthesis. (a)** This denture was firmly retained with the cast clasps. **(b)** Support with this part against sinkage of the prosthesis (arrows).

Two months later, because the H-shaped miniplate was stable (Figure 6a,c), a second prosthesis was fabricated (Figure 7a,b). This prosthesis had three cast clasps bilaterally to retain the prosthesis by the three remaining molars. Anteriorly, this prosthesis was supported by the miniplate so that it did not sink superiorly. The miniplate prevented the counterclockwise rotation of the prosthesis when biting. These structures allowed for easier insertion and removal of the denture. The patient’s ability to ingest ordinary food also improved.

The entire surgical margin was sufficiently free from the tumor. The dissected surgical specimen of the neck showed one lymph node metastasis in the ipsilateral submandibular region. Extranodal spread from the lymph node was not recognized. Although adjuvant radiotherapy was recommended, the patient declined additional treatment.

Reconstruction of the maxilla with a vascularized bony flap and insertion of dental implants for definitive dental rehabilitation were suggested for this patient 1 year postoperatively. However, the patient declined our suggestion because he was very satisfied with the second prosthesis on the H-shaped miniplate.

**Figure 8:**
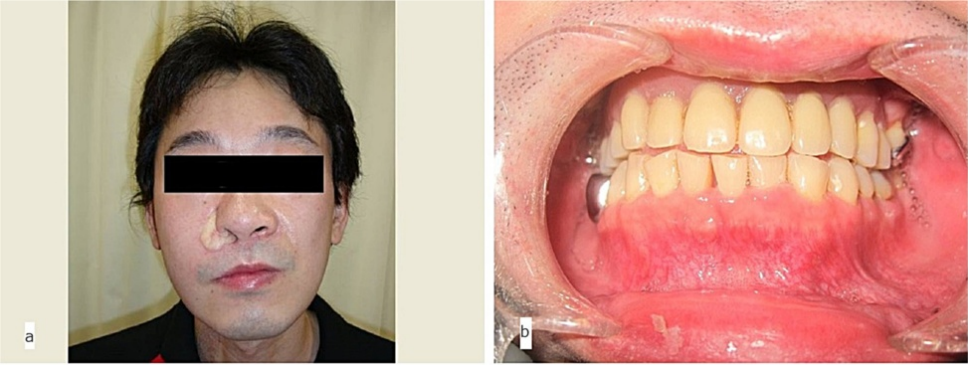
**Postoperative view. (a)** Frontal view 2 years 6 months postoperatively. **(b)** Frontal oral view with definitive epithesis.

The postoperative course was uneventful. Neither recurrence nor metastasis was found 2 years 6 months postoperatively (Figure 8a,b). The miniplate has not fractured and has remained stable. The three remaining teeth are sound, and neither dental caries nor periodontitis has developed. The patient’s masticatory, deglutitive, and speech functions are adequate. He is satisfied with both the dental rehabilitation and facial aesthetics.

## Conclusions

Maxillary defects caused by cancer ablative surgery are commonly reconstructed with prostheses. Good functional results can reportedly be attained with obturator prostheses [1-4]. However, the further the resected region extends the less stabile the prosthesis becomes because of insufficient bone and tooth support for the denture. Overloading of the remaining teeth by the prosthesis leads to tooth loss, and the stability of the prosthesis deteriorates. This leads to inadequate masticatory function, deglutition, and speech and objectionable cosmetics.

In the present case, the stability of the immediate and definitive prosthesis seemed insufficient because of the large anterior maxillary defect and only three remaining teeth (maxillary right second molar and maxillary left first and second molars). Therefore, an H-shaped miniplate was applied to the hard palate to support the prosthesis. We selected this miniplate because this method is extremely easy and feasible and is thought to effectively prevent superior sinkage of the prosthesis, which rotates counterclockwise when biting. The combination of orthodontic elastics with the miniplate improved the stability of the prefabricated prosthesis. This structure retained the ointment gauze covering the wounds and maintained the facial contour postoperatively. Contracture deformity was also prevented by immediate insertion of the prefabricated prosthesis.

The patient could ingest a soft diet with the prefabricated prosthesis, and the diet was close to normal with the definitive prosthesis. We assessed the masticatory efficiency of the definitive prosthesis with the Japanese version of the Oral Health Impact Profile (OHIP-J) [5]. This questionnaire comprises 25 food items for evaluation of the masticatory ability. The patient could easily eat all of the items with the exception of four foods, which he did not eat because he disliked them. These results suggest that he had a sufficient masticatory ability with this definitive prosthesis.

Loss of the abutment teeth for the prosthesis is often experienced clinically. Overloading of the abutment teeth is thought to be the cause of the tooth loss. Especially in larger maxillary defects, the overloading of the remaining teeth with the larger prosthesis will be more severe. In the present patient, because the vertical overload in the direction of tooth extraction was decreased by the miniplate, the risk of tooth loss may have been lower. Further cases are needed to evaluate this issue.

Undercutting of the soft tissues is generally utilized for retention of the prosthesis in cases of large maxillary defects. Prosthesis-induced stomatitis is not rare because of prosthetic instability. Prosthesis-induced stomatitis was not seen in our case. The stability of the prosthesis with the miniplate in the palate was thought to be effective for the prevention of prosthetic stomatitis.

In cases of miniplate application in the palate, the miniplate should not be firmly attached to the prosthesis (e.g., using a magnetic attachment) because this increases the possibility of miniplate fracture. Maintenance of only slight contact between the miniplate and the prosthesis is quite important (see Figure 7b).

Mini dental implants are becoming increasingly popular in dental care [6,7]. The mini dental implant may have been effective for the present patient. In this case, we selected a miniplate for the reasons described above. We believe that the herein-described miniplate application more effectively prevented superior sinkage of the prosthesis, which rotates counterclockwise when biting. However, the combination of this miniplate and mini dental implants could have been more effective for the prosthesis. This combination may be considered for dental rehabilitation in patients with large anterior maxillary defects.

Immediate reconstruction of the maxilla with a vascularized bony flap and insertion of a dental implant is also widely performed in dental rehabilitation [8,9]. Moreno et al. reported that moderately sized maxillectomy defects involving the palate can be successfully treated with either an obturator or free flap reconstruction; however, extensive defects have a better functional outcome with free flaps [10]. In our case, bony reconstruction and insertion of a dental implant should have been performed because the maxillary defect was relatively extensive and our patient was relatively young. However, bony reconstruction of the maxilla was not performed, and only soft tissue was immediately reconstructed with a partially double-folded free radial forearm flap for maintenance of his facial aesthetics. This was done because the malignant tumor grew rapidly and aggressively. We planned to first control the tumor, then perform delayed reconstruction of the maxilla with a vascularized bony flap when neither recurrence nor metastasis was found postoperatively. Postoperative local observation is very easy for open surgical wounds [11]. Soft tissue reconstruction and placement of the prosthesis were thought to be adequate for maintenance of the facial aesthetics and temporary dental rehabilitation in the present patient.

Bony reconstruction with a free flap and insertion of dental implants for definitive dental rehabilitation was suggested for the patient 1year postoperatively when neither recurrence nor metastasis was found. However, the patient declined our suggestion because he was satisfied with both the facial aesthetics and dental rehabilitation with the obturator on the miniplate. If any fault of the miniplate occurs, secondary reconstruction of the maxilla with a free flap will be actively recommended. If less invasive surgery is requested, we will insert mini dental implants and zygomatic implants. In our department, reconstruction of maxillary defects is performed with a prosthesis as first-line therapy because we have frequently experienced flap descensus secondary to prosthesis instability and flap contamination. However, bony reconstruction and insertion of dental implants for definitive dental reconstruction should be performed in the future in the present case considering the patient’s age and the possibility of a fault with the miniplate.

The application described herein is associated with a risk of screw loosening, miniplate fracture, and so on. Therefore, quantitative strength analysis may be needed. However, considering the fact that this patient could ingest a diet close to normal with this prosthesis for 2 years 6 months without troubling issues, the strength is thought to be sufficient for temporary dental rehabilitation. Unfortunately, this case represents the first trial of this method, and quantitative strength analysis could not be performed; thus, additional cases are needed for further assessment. Three-dimensional finite element analysis may also be needed in the future.

This technique is reasonable and feasible, but it may not be applicable to all patients with large anterior maxillary defects. The requirements for this technique are as follows: 1) Sufficient palatine bone is present for fixation of the H-shaped miniplate. 2) Some remaining teeth are present to stabilize the prosthesis.

In summary, application of titanium miniplates in the palate for treatment of large anterior maxillary defects is notable for its role in temporary dental rehabilitation.

### Consent

Written informed consent was obtained from the patient for publication of this case report and any accompanying images. A copy of the written consent is available for review by the Editor-in-Chief of this journal.

## Competing interests

The authors declare that they have no competing interests.

## Authors’ contributions

SI , NKand HT performed the operation and monitored the patient. TK, HY, and MI assisted in the surgery. All authors read and approved the final manuscript.
